# Fetal Safety in MRI During Pregnancy: A Comprehensive Review

**DOI:** 10.3390/diagnostics15020208

**Published:** 2025-01-17

**Authors:** Gal Puris, Angela Chetrit, Eldad Katorza

**Affiliations:** 1 Arrow Program for Medical Research Education, Sheba Medical Center, Tel Hashomer, Ramat Gan 52621, Israel; eldad.katorza@sheba.health.gov.il; 2 Gertner Institute for Epidemiology and Health Policy Research, Chaim Sheba Medical Center, Tel Hashomer, Ramat Gan 52621, Israel; 3 Department of Obstetrics and Gynecology, Sheba Medical Center, Tel Hashomer, Ramat Gan 52621, Israel

**Keywords:** MRI, 3 T MRI, pregnancy, safety

## Abstract

As medical imaging continues to expand, concerns about the potential risks of ionizing radiation to the developing fetus have led to a preference for non-radiation-based alternatives such as ultrasonography and fetal MRI. This review examines the current evidence on the safety of MRI during pregnancy, with a focus on 3 T MRI and contrast agents, aiming to provide a comprehensive synthesis that informs clinical decision-making, ensures fetal safety and supports the safe use of all available modalities that could impact management. We conducted a comprehensive review of studies from 2000 to 2024 on MRI safety during pregnancy, focusing on 3 T MRI and gadolinium use. The review included peer-reviewed articles and large database studies, summarizing key findings and identifying areas for further research. Fetal MRI, used alongside ultrasound, enhances diagnostic accuracy for fetal anomalies, particularly in the brain, thorax, gastrointestinal and genitourinary systems, with no conclusive evidence of adverse effects on fetal development. While theoretical risks such as tissue heating and acoustic damage exist, studies show no significant harm at 1.5 T or 3 T, though caution is still advised in the first trimester. Regarding gadolinium-based contrast agents, the evidence is conflicting: while some studies suggest risks such as stillbirth and rheumatological conditions, animal studies show minimal fetal retention and no significant toxicity, and later clinical research has not substantiated these risks. The existing literature on fetal MRI is encouraging, suggesting minimal risks; however, further investigation through larger, prospective and long-term follow-up studies is essential to comprehensively determine its safety and late effects.

## 1. Introduction

As the use of medical imaging continues to grow, there is increasing concern about the potential effects of diagnostic procedures on the developing fetus [1]. Ionizing radiation, commonly used in various imaging techniques, is well recognized for its risks to fetal development, prompting clinicians to seek alternative methods with minimal or no radiation exposure. Among these alternatives, ultrasonography and magnetic resonance imaging (MRI) are frequently preferred due to their safety profiles [2,3]. Fetal MRI is gaining prominence due to its advantages [4], and it provides high-resolution images of the fetal brain and detailed views of structures such as the lungs, liver, kidneys and bowel. Its superior soft tissue contrast and large field of view make it particularly valuable for assessing complex anomalies.

Although MRI is widely regarded as safe for fetal imaging, several theoretical concerns regarding the use of MRI during pregnancy may contribute to unnecessary anxiety among patients, their families and healthcare professionals, potentially hindering its broader application [5]. Concerns about fetal safety with the use of MRI relate to various issues: risk of slow growth in utero, premature birth and potential cochlear dysfunction after exposure to acoustic noise. The challenge of evaluating long-term risks is exacerbated by the limited availability of large-scale, prospective studies with extended follow-up periods, as designing such trials is often impractical. Much of the current evidence is extrapolated from smaller case studies and animal models [6]. The most robust evidence available comes from large public health database studies, which have helped mitigate some safety concerns associated with MRI during pregnancy [7]. Technological advancements, particularly the adoption of higher field strengths such as 3 Tesla (3 T) MRI systems, have led to improvements in image quality and diagnostic performance through enhanced signal-to-noise ratios and spatial resolution. While these advancements offer significant benefits [8] for maternal and fetal imaging, the safety profile of 3 T MRI compared to 1.5 T MRI remains inadequately studied [9,10].

Given the current gaps in knowledge, further research is essential to fully understand the short- and long-term safety implications of MRI, particularly in early pregnancy, with gadolinium contrast or with emerging MRI modalities. Clinicians require more comprehensive data to make informed decisions about the use of MRI in pregnant patients, ensuring both the safety of the developing fetus and the avoidance of missing crucial diagnostic information if the procedure is deemed safe.

This review aims to gather and assess the existing literature on the short- and long-term effects of MRI exposure during pregnancy, focusing specifically on 3 T MRI and the use of contrast agents. Our objective is to provide a thorough synthesis of current knowledge to inform clinical decision-making, ensuring that healthcare providers seize the opportunity to accurately diagnose conditions and implement optimal management strategies that safeguard the health of both mother and fetus.

## 2. Materials and Methods

We conducted a comprehensive review of the literature by searching electronic databases, including PubMed, MEDLINE, Embase and Cochrane Library, using keywords related to “MRI safety during pregnancy”, “MRI effects on fetus”, “3 T MRI modalities in pregnancy” “MRI versus Ultrasound in pregnancy” and “contrast MRI during pregnancy” from January 2000 to August 2024. Studies were selected based on their focus on the safety and efficacy of MRI during pregnancy, specifically regarding 3 T MRI modalities and gadolinium use. Inclusion criteria encompassed peer-reviewed articles, systematic reviews and large public health database studies, excluding small case reports. The findings were synthesized to summarize current knowledge, highlight key trends and identify areas for further research.

## 3. Results

All studies examining outcomes after fetal MRI exposure included in the review are summarized in Table 1.

### 3.1. MRI Versus Ultrasound

Ultrasound (US) remains the primary imaging modality for evaluating pregnancy-related pathologies; however, fetal MRI has advantages and specific indications as an adjunct to US. Unlike US, fetal MRI is not substantially hindered by maternal obesity, fetal positioning or oligohydramnios, and it provides superior visualization of specific fetal structures [11,12]. Owing to its enhanced soft tissue contrast resolution, MRI can delineate specific fetal organs, such as the lungs, liver, kidneys and intestines [13] and provide additional anatomical information in comparison to US. Levine et al. [14] collected a cohort of 83 women with 90 fetuses that went through 91 ultrasonographic and MRI examinations of the fetal central nervous system (CNS). MRI findings led to changed diagnoses in 26 (40%) of 66 fetuses with abnormal confirmatory sonograms. They concluded that when a CNS anomaly is detected by sonography or suspected on ultrasound, MRI findings might lead to altered diagnosis and patient counseling. Griffiths et al. [15] performed the largest multicenter, prospective and appropriately powered study in the UK, with women carrying a fetus suspected of having a brain anomaly on ultrasound had in utero MRI performed within 14 days of ultrasound. The results were evaluated by two separate panels to assess diagnostic accuracy and confidence through comparison with outcome diagnoses. Additionally, the impact of MRI on diagnosis, prognosis and clinical management was examined, along with an evaluation of patient acceptability. The overall diagnostic accuracy was 68% for ultrasound and 93% for MRI (difference 25%, 95% CI 21–29). MRI provided additional diagnostic information in 387 (49%) of 783 cases, changed prognostic information in at least 157 (20%) and led to changes in clinical management in more than one in three cases. MRI demonstrated high patient acceptability, with the majority of women indicating they would choose an MRI study if a future pregnancy were affected by a fetal brain abnormality. Kul et al. [16] compared prenatal US and MRI diagnoses of 151 fetuses with respect to postnatal diagnoses. The contribution rates of MRI to US in the diagnosis of fetal anomalies were 55% for the central nervous system (*p* < 0.001), 44% for thorax (*p* = 0.016), 38% for gastrointestinal system (GIS) (*p* = 0.031) and 29% for genitourinary system (GUS) (*p* = 0.003) anomalies. They concluded MRI imaging can be used as an adjunct to US in the prenatal diagnosis of fetal anomalies of not only the CNS but also the non-CNS origin especially those involving the GIS, GUS and thorax. Furthermore, MRI offers the advantage of multiplanar imaging and a wide field of view, enabling comprehensive assessment of fetuses with large or intricate anomalies and facilitating the evaluation of lesions within the context of the entire fetal anatomy. In a variety of brain and spine disorders, prenatal MRI imaging can delineate and characterize the abnormality, thereby enhancing diagnostic precision and aiding in the planning of postnatal surgical interventions and management.

In a study by Elka et al., 14 out of 24 fetuses with CNS abnormalities identified through prenatal MRI underwent surgery based on the imaging findings [17]. The diagnostic utility of fetal MRI is limited during early gestation due to the small size of the fetus and the challenges posed by fetal motion [18]. A comprehensive review of fetal MRI technique and the use of different sequences is detailed in different guidelines and studies [4,19,20].

### 3.2. Short Term Outcome of Fetal MRI

The American College of Radiology and Society for Pediatric Radiology practice parameter for the safe and optimal performance of fetal MRI, revised in 2024, cited data from various studies, which showed no conclusive deleterious effects of 1.5 T or 3 T MRI on the developing fetus. Currently published safety data regarding 3 T MRI are limited. To our knowledge, existing studies have shown no evidence of short-term outcomes including fetal growth, birth weight percentiles and neonatal hearing compared with findings in neonates not exposed to 1.5 T MRI. Most of them had limitations, such as the small sample size, lack of a control group and a short follow-up period [21,22].

Choi et al. [23] reported a case series of 15 women inadvertently exposed to magnetic resonance imaging with a 1.5 T in the first trimester of pregnancy. Patients were prospectively followed up until the completion of their pregnancy, 15 babies were born alive and none with abnormalities that were considered by the authors related to MRI exposure. Strizek et al. [24] conducted a retrospective case–control study with a group of 751 neonates exposed to 1.5 T MRI imaging in utero and a group of control subjects comprising 10,042 non-exposed neonates. The rate of hearing impairment or deafness was found to be 0% in the neonates in the exposed group and was not inferior to that in the non-exposed group (0.34%, *p* < 0.05). There was no between-group difference in birth weight percentiles (50.6% for exposed versus 48.4% for non-exposed; *p* = 0.22). In a large retrospective database study conducted by Ray et al. [25], the risk of stillbirth or neonatal death within 28 days of birth and any congenital anomaly, neoplasm and hearing or vision loss was evaluated from birth to age 4 years in children with first-trimester MRI exposure. Overall, the incidence rate of all outcomes was not significantly higher in the offspring of exposed women, but upon restricting MRI exposure to between 5 and 10 weeks of gestation, the risk of vision loss was higher, with an adjusted HR of 2.28 (95% CI, 1.09–4.77). We have not found similar studies examining this outcome. Jaimes et al. [21] examined if there was an increased prevalence of congenital hearing loss in 62 neonates who had had a 3 T prenatal MRI versus a random control group who had had it at 1.5 T. The fail rates of transient otoacoustic emissions test for the 1.5 T and 3 T groups were 9.7% and 6.5%, respectively, and for the auditory brainstem response test were 3.2% and 1.6%, respectively. There was no significant difference in the fail rate of either test between groups. In a study published in 2019 by Chartier et al. [22] including consecutively born healthy neonates exposed in utero to 3 T MRI for maternal or fetal indications and randomly matched by the birth date of healthy control neonates who had not been exposed to MRI, no significant difference in the mean birth weight between the MRI-exposed (3398 g) and control (3510 g) neonates (*p* = 0.06) was found. No adverse effect with regard to neonatal hearing was found in this study.

### 3.3. Long-Term Outcome of Fetal MRI

The association between MRI exposure during pregnancy and behavior and developmental disorders has been examined in a few studies using different methodology in terms of the type of questionnaires used, the outcomes measured and the duration of follow-up. All of these studies, however, consistently found no difference between individuals exposed to fetal MRI and those unexposed [7,26,27,28].

A few studies investigated long-term effects of MRI imaging antenatally. Zvi et al. [7] conducted a historical prospective cohort study to examine long-term neurodevelopmental outcomes of 131 children aged 2.5 to 6 years, exposed to 1.5 T non-contrast MRI imaging as fetuses. No difference was identified in the Vineland-II Adaptive Behavior Scale composite score between the children of the study and control groups (mean, 110.79 versus 108.18; *p* = 0.098). Differences were also not observed between the children of the two groups in specific questionnaire domains. A study of 72 toddlers exposed to MRI as fetuses examined adaptive behavior at age two using a parent telephone questionnaire [29]. The study found that all toddlers were within the normal range for the behaviors examined. Another study that followed 31 children aged 5 to 7 years, who underwent repeated MRI scans (up to 10 per year) for a decade, reported that no cognitive changes in IQ measures and language ability were found over the years [27].

The effects of 1.5 T versus 3 T magnetic resonance imaging (MRI) on postnatal neurodevelopmental outcomes were evaluated in a study of 100 fetuses with left-sided congenital diaphragmatic hernia (n = 75, 1.5 T; n = 25 3 T). Children were evaluated at 24 months using the Bayley Scales of Infant Development III, and no significant difference in neurodevelopmental outcomes was observed between the 1.5 T and 3 T groups [28].

To the best of our knowledge, no studies have been published to date on the long-term effects of exposure to 3 T MRI during pregnancy.

### 3.4. Non-Contrast 3 T MRI During Pregnancy

The theoretical risks associated with fetal exposure to MRI during pregnancy include potential tissue heating that may lead to miscarriage or injury to organogenesis in the first trimester and the possibility of acoustic damage due to exposure to the magnetic field [30]. The transition from 1.5 to 3.0 T in fetal MRI brings safety concerns associated with the higher magnetic field strength and radiofrequency power [31].

The Canadian Association of Radiologists stated that most studies have shown no adverse outcomes attributable to MRI in any trimester at either 1.5 T or 3 T; however, as an act of caution, 1.5 T is preferred in the first trimester if both field strengths are available [30].

We will address the existing knowledge regarding each of the potential negative effects when using 3 T modalities. A prospective observational study conducted by Bouyssi-Kobar et al. [26] included 72 healthy pregnant women, who underwent fetal MRI at a mean gestational age of 30.5 ± 3.1 weeks. The mean age at follow-up testing was 24.5 ± 6.7 months. All children had age-appropriate scores in the communication, daily living, socialization and motor skills subdomains of the Vineland Adaptive Behavior Scale. All children passed their newborn otoacoustic emission tests and had normal hearing at preschool age. Furthermore, MRI study duration and exposure time to radio frequency waves and SSFSE sequences were not associated with adverse functional outcomes or hearing impairment.

- **Tissue heating:**

Exposure to radiofrequency pulses can potentially lead to deposition of energy in body tissues in the form of heat. Because the fetal temperature cannot be measured directly, the specific absorption ratio (SAR) is used to estimate the absorbed energy in the maternal tissue (W/kg) assuming this would be the maximal fetal temperature change. Clinical concerns have been raised regarding the use of 3 T MRI scanners compared to 1.5 T MRI scanners, primarily due to the potential for higher SAR and the associated risk of fetal heating at 3 T [31].

In a study on pregnant miniature pigs [32], the use of 3 T magnets for diagnostic MRI with normal SAR regimens showed maximal temperature increases of 1 °C if imaging time is kept below 30 min. Longer imaging time, especially with high-SAR regimens, can lead to an increase of 2.5 °C. Significant differences in thermoregulation and thermoneutral ambient temperatures make direct extrapolation of animal data to humans challenging. Nonetheless, the FDA and the International Electrotechnical Commission (IEC) [33] used very conservative estimations and set strict limits with an upward SAR limit under normal operating mode for the general population and pregnant patients specifically. Operation in normal mode limits the rise of body temperature to 0.5 °C.

Barrera et al. [9] performed a retrospective study of 93,764 MRI sequences—81,535 performed at 1.5 T and 12,229 preformed at 3 T. The examinations in this study were found to have equivalent energy metrics except specific sequences (two-dimensional T1-weighted spoiled gradient-echo and three-dimensional steady-state free precession). They concluded that other than these specific sequences that may require modification to keep the energy delivered to the patient at the known accepted levels, the 3 T examinations do not exceed energy level limitations.

Another recent study carried out in California [34] estimated the fetal brain temperature before and after T2-weighted SSFSE images by proton resonance frequency (PRF) thermometry and compared to the estimated temperature in the gluteal muscle of the mother. For the 32 participants used in the analysis, 17 with cardiac abnormalities and 15 healthy controls, estimated relative temperature changes of the fetal brains were minimal. These findings support that fetal brain imaging at 3 T is within FDA limits and safe.

- **Acoustic damage:**

Acoustic noise in MRI primarily arises from Lorentz forces [35], which are produced by rapid changes in current within the gradient coils. These forces are directly related to the strength of the main magnetic field and the gradient current. Noise levels also depend on the machine design, sequence and protocol used.

Large retrospective studies of fetuses exposed to 1.5 T MRI during the first trimester, including a cohort of 1737 cases [25], found no significant differences in auditory outcomes compared to a control group of approximately 1.4 million non-exposed fetuses, with no hearing impairments detected at birth or during a 4-year follow-up. Similarly, investigations of fetuses scanned in the second and third trimesters, including a retrospective case–control study [24] of 751 neonates exposed to MRI imaging in utero, showed no adverse effects on fetal hearing after birth. These findings are in line with smaller studies [26,36] that demonstrate similar results in follow-up assessments at 3 months. Studies evaluating short and long-term effects of 3 T MRI on neonatal hearing are beginning to emerge, but research in this area remains limited. In 2019, Chartier et al. [22] conducted a single-center retrospective case–control study evaluating the effects of clinical 3 T MRI during any trimester of pregnancy on neonatal hearing. They found no significant difference in the prevalence of hearing impairment (*p* = 0.55) between the MRI-exposed (0% [0/81]) and control (1.8% [3/162]) groups at any gestational age of MRI exposure, 14 of the exposures occurred at the 1st trimester. Published in 2019, Jaimes et al. [21] evaluated the impact of 1.5 T versus 3 T MRI on neonatal auditory function in the second and third trimesters, with 62 neonates in each cohort. They observed no statistically significant differences in the failure rates of the transient otoacoustic emissions test (9.7% for 1.5 T versus 6.5% for 3 T, *p* = 0.74) or the auditory brainstem response test (3.2% for 1.5 T versus 1.6% for 3 T, *p* = 0.80). To our knowledge, there are currently no published large-scale or long-term studies on this subject.

### 3.5. Gadolinium-Based Contrast Agent (GBCA) MRI During Pregnancy

Gadolinium-based contrast agents (GBCAs) complexed with chelators are used for specific indications in order to enhance the clarity and detection of images, improving diagnoses [37]. The chelators do not completely prevent patient exposure to gadolinium, and there are known adverse effects to the use of these agents. The short-term risk is categorized as hypersensitivity-related reactions and are not specific to pregnant patients. Treatment in this situation should take specific measures for fetal optimal care [38]. Long-term risks include nephrogenic systemic fibrosis (NSF) and potentially retained gadolinium in fetal tissues. NSF is a rare condition characterized by fibrosis and organ failure that occurs in patients with impaired renal function who have been exposed to gadolinium-based contrast agents during magnetic resonance imaging. Since 2006, the FDA issued several warnings advising significant caution when using GBCAs in patients with known renal disease.

Multiple studies have demonstrated the deposition of gadolinium from GBCAs in various tissues, particularly the brain, bones and skin. Gibby et al. [39] and White et al. [40] observed gadolinium retention in femoral bone tissue following hip replacement surgeries in patients who received IV GBCAs prior to surgery. Darrah et al. [41] further confirmed prolonged gadolinium retention in bone, noting higher concentrations in trabecular bones. However, of greater concern is the accumulation of gadolinium in the brain. Autopsy studies, including those by McDonald et al. [42,43,44], have revealed gadolinium deposition in the brains of patients with normal renal function and intact blood–brain barrier, with highest concentrations in the globus pallidus and dentate nuclei. These deposits were dose-dependent and persisted in small concentrations for years after the last contrast administration. It is important to mention they found no evidence of gadolinium-mediated histological changes to suggest a toxic effect, and no clinical long-term adverse effects have been proved.

Regarding GBCAs in MRI during pregnancy specifically, two principal areas have been studied: animal studies examining the retention of contrast agents in fetal tissues and exposure effects on fetal development and clinical retrospective cohorts searching for association with adverse pregnancy outcomes. There is only one large cohort study [25] that suggests the use of gadolinium-based contrast agents (GBCAs) during MRI at any point in pregnancy may be associated with a slightly increased risk of stillbirth or neonatal death, as well as a broader range of rheumatological, inflammatory or infiltrative skin conditions. However, due to the limited data available [45,46], the safety profile of GBCAs for the fetus remains inconclusive [47]. Further research is needed to clarify these findings and better define the role of GBCAs during pregnancy.

- **Animal studies:**

Gadolinium contrast media have been shown in the placenta following intravenous contrast administration to the mother in animals in various studies [48]. In 2015, Karen Oh et al. [49] conducted a study on 14 Gravid Japanese macaques. The study included injection of these primates with IV Gadoteridol and delivery by means of cesarean section within 24 h. Gadolinium chelate levels in the placenta, fetal tissues and amniotic fluid were obtained. Gadoteridol was present in the fetoplacental circulation at much lower quantities than in the mother. Minimal amounts were detected in the fetal kidney, amniotic fluid and placenta and decreased significantly within hours. Similar findings were demonstrated in a study by Prola-Netto et al. [50] in 2017. To date, no symptoms have been observed following retained gadolinium, thus the clinical significance remains uncertain [51]. A meta-analysis of 18 animal studies from 1988 to 2012 examined the effect of MRI during pregnancy on fertility and development outcomes of the offspring and found no evidence of risks for all outcomes examined [52].

Some previous animal studies have shown potential fetal toxic effects of contrast media, but these effects have never been observed when using approved contrast media in the doses used in human imaging protocols [48].

- **Clinical retrospective cohorts:**

In 2016, a population-based cohort study [25] by Ray et al. reported that GBCA-MRI exposure during pregnancy was associated with an increased risk of stillbirth or neonatal death (an adjusted RR of 3.70 for, although this finding was based on only seven events in the gadolinium MRI group) and childhood rheumatological, inflammatory or infiltrative skin conditions (an adjusted HR of 1.36). In additional analyses of gadolinium MRI, only first-trimester exposure was associated with a higher risk of any rheumatological, inflammatory or infiltrative skin condition. Limitations of this study included insufficient sample size to support a statistical comparison of contrast MRI versus non-contrast MRI, inadequate control for the reason MRI was administered and bundling of rare outcomes.

A subsequent study [53] conducted in 2023 by the FDA/CDER in collaboration with researchers at the University of Florida constructed a retrospective cohort of >11 million Medicaid-covered pregnancies to evaluate the association between prenatal magnetic resonance imaging exposure with and without gadolinium-based contrast agents and fetal and neonatal death (primary endpoint) and neonatal intensive care unit admissions (secondary endpoint). The findings revealed that the risk for pregnancies exposed to GBCAs was similar to that of those exposed to MRI without contrast. No significant increase in NICU admissions was observed in newborns exposed to GBCAs. Sensitivity analyses confirmed the stability of these results. While the study did not address subacute or chronic outcomes, its findings contribute valuable information to the safety profile of GBCA-MRI with respect to acute effects. Further research is needed to explore the potential impact of GBCA exposure on long-term and chronic outcomes.

**Table 1:** Fetal safety in MRI during pregnancy: summary of the literature.

Name of Article	Journal	Type of Study	Publication Year	Study Population	MRI Indication	Exposure	Outcome Checked (Development, Obstetrical, Deformities)	Findings	Limitations
Absence of harmful effects of magnetic resonance exposure at 1.5 T in utero during the third trimester of pregnancy: a follow-up study [36]	Magnetic resonance imaging	follow-up study	2004	35 children between 1 and 3 years of age and 9 children between 8 and 9 years of age		Third trimester, 1.5 T	Results of a neurological examination at 3 months, their medical documentary with emphasis on eye and ear functioning and questionnaires answered by their mothers were collected and evaluated.	No abnormalities were observed in 37 of the 41 children included in this study. In four children, there are deficits that were considered to be unrelated to MRI exposure.	1. Small cohort. 2. Follow-up study with no control group.
A case series of 15 women inadvertently exposed to magnetic resonance imaging in the first trimester of pregnancy [23]	Journal of Obstetrics and Gynaecology	Case series	2015	15 exposed	Mother indication	First trimester, 1.5 T	Abnormalities until birth	Fifteen babies born alive. Of them, one baby was born with the left kidney not visualized by ultrasound examination and another one with an overlapping toe in the right foot. None of these abnormalities were considered by the authors related to MRI exposure.	Case series
Safety of MR Imaging at 1.5 T in Fetuses: A Retrospective Case-Control Study of Birth Weights and the Effects of Acoustic Noise. [24]	Radiology	Retrospective case–control study	2015	751 neonates exposed to MR imaging in utero, 10,042 control non-exposed neonates	Maternal/fetal indications	All pregnancy, 1.5 T	Effects of exposure to routine magnetic resonance (MR) imaging at 1.5 T during pregnancy on fetal growth and neonatal hearing function in relation to the dose and timing	No between-group difference in birth weight percentiles or hearing impairment.	1. Retrospective. 2. Many patients had short exposure to MR imaging. 3. Only healthy newborns.
Fetal magnetic resonance imaging: exposure times and functional outcomes at preschool age [26]	Pediatric Radiology	Prospective observational study	2015	72 exposed		2,3rd trimester, 1.5 T	Functional outcomes were assessed using the Vineland Adaptive Behavior Scale (VABS), otoacoustic emission test and hearing at preschool age	MRI study duration and exposure time to radio frequency waves and SSFSE sequences were not associated with adverse functional outcomes or hearing impairment.	1. Follow-up for hearing based on parents’ report. 2. Functional assessments through telephone interview only.
Association Between MRI Exposure During Pregnancy and Fetal and Childhood Outcomes [25]	JAMA	Retrospective cohort study	2016	1st trimester MRI group—1737. Control—1418, 451. Gadolinium MRI group— 397. Control—1,418,451.	-	1st trimester cohort—MRI. All pregnancy cohort—GBCAs.	For 1st-trimester MRI (cohort 1), five study outcomes diagnosed before age 4 years were assessed: 1. Stillbirth after 20 weeks’ gestation or neonatal death before 28 days after birth; 2. Any congenital anomaly, excluding children with a concomitant chromosomal disorder; 3. Neoplasm; 4. Vision loss; 5. Hearing loss. For gadolinium-enhanced MRI during pregnancy (cohort 2), a specific NSF-like outcome of a connective tissue or skin disease was evaluated, diagnosed from birth to age 4 years, a broader outcome of any diagnosed rheumatological, inflammatory or infiltrative skin conditions were assessed.	Exposure to MRI during the first trimester of pregnancy compared with non-exposure was not associated with an increased risk of harm to the fetus or in early childhood. Gadolinium MRI at any time during pregnancy was associated with an increased risk of a broad set of rheumatological, inflammatory or infiltrative skin conditions and for stillbirth or neonatal death.	1. First-trimester cohort—analyses were underpowered to assess uncommon outcomes. 2. Several models with different outcomes were created—type 1 statistical error. 3. Risk posed by 1st-trimester MRI may have been underestimated (all pregnancies ending before 21 weeks’ gestation were excluded.) 4. No data regarding the indication of MRI. 5. Large proportion of children not followed up for the full period of study.
Does 3 T fetal MRI induce adverse acoustic effects in the neonate? A preliminary study comparing postnatal auditory test performance of fetuses scanned at 1.5 and 3 T [21]	Pediatric Radiology	Retrospective case–control study	2019	62 exposed, 62 control	Fetal indications	3rd trimester, 1.5 T/3 T	The pass/fail rate of the transient otoacoustic emissions test and auditory brainstem response test	No significant difference in the fail rate of either test between groups.	1. Retrospective 2. Small sample size 3. Selection bias—3 T MRI median week fetal exposure 5 weeks older than 1.5 T. 4. A high rate of FN in the neonatal hearing screening test with no continuation of follow-up.
The Safety of Maternal and Fetal MRI at 3 T [22]	AJR	Retrospective case–control study	2019	81 exposed, 162 control	Maternal/fetal indications	All pregnancy, 3 T	Fetal growth and neonatal hearing	No significant difference in mean birth weight or prevalence of hearing impairment between groups.	1. Retrospective 2. Small sample size 3. Only healthy neonates included. 4. Limited outcomes examined. 5. Seventy-four percent of MRI were for maternal indications—may have shorter imaging times and not directly image the fetus.
Fetal Exposure to MR Imaging: Long-Term Neurodevelopmental Outcome [7]	AJNR	Historical prospective cohort study	2020	131 exposed women, 771 control	Maternal/fetal indications	All pregnancy, 1.5 T	Long-term neurodevelopmental outcomes	No difference was identified in the Vineland-II Adaptive Behavior Scale composite score.	1. Small number of women were exposed to MRI during the 1st trimester—limited assessment of MRI exposure effects during this trimester. 2. In this study, 35.3% of MRI indication were for the fetal CNS—may cause a selection bias.
Risk of fetal or neonatal death or neonatal intensive care unit admission associated with gadolinium magnetic resonance imaging exposure during pregnancy [53]	American Journal of Obstetrics and Gynecology	Retrospective cohort study	2023	782 GBCA, 5209 non-GBCA	-	All pregnancy, GBCA	Death or neonatal morbidity requiring NICU admission.	Among 5991 qualifying pregnancies—11 fetal or neonatal deaths in the gadolinium-based contrast agent magnetic resonance imaging group (1.4%) and 73 in the non-gadolinium-based contrast agent magnetic resonance imaging group (1.4%) with an adjusted relative risk of 0.73. The NICU admission adjusted relative risk was 1.03.	1. Limited generalizability: The study population consisted of women covered by Medicaid, which might not be representative of the entire pregnant population. 2. Estimation of gestational age. 3. Most MRIs with GBCAs occurred in the first trimester. 4. Pregnant women who received GBCA-enhanced MRIs might have underlying medical conditions that could also increase the risk of fetal or neonatal death. The study tried to address this by excluding certain diagnoses and using propensity score weighting, but residual confounding is still possible.
Effects of 1.5 T versus 3 T magnetic resonance imaging in fetuses: is there a difference in postnatal neurodevelopmental outcome? Evaluation in a fetal population with left-sided congenital diaphragmatic hernia [28]	Pediatric Radiology	Retrospective review	2023	Seventy-five fetuses with left congenital diaphragmatic hernia, scanned at 1.5 T. Twenty-five fetuses with left congenital diaphragmatic hernia, scanned at 3 T.	Left congenital diaphragmatic hernia	All pregnancy, 1.5 T/3 T	Neurodevelopmental outcomes were assessed using the Bayley Scales of Infant Development, 3rd Edition (BSID-III).	No statistical differences in mean BSID-III cognitive, language and motor composite scores, subscales scores or risk of abnormal neuromuscular exam.	1. Retrospective study design. 2. Relatively small sample size. 3. Selection bias: The study population was limited to fetuses with left-sided congenital diaphragmatic hernia.

## 4. Discussion

The safety of MRI during pregnancy has been a subject of concern, primarily due to theoretical risks such as fetal tissue heating, acoustic damage and the potential effects of gadolinium-based contrast agents (GBCAs). It is crucial to remember that the use of GBCAs during pregnancy should be considered carefully and only when benefits outweigh potential risks. Although current evidence largely supports the safety of MRI, particularly with non-contrast 1.5 T and 3 T modalities, most studies are retrospective and remain limited by small sample sizes and short follow-up periods.

Existing research shows no significant adverse short-term outcomes, including fetal growth, birth weight or neonatal hearing, with both 1.5 T and 3 T MRI. However, there remains a gap in understanding the long-term neurodevelopmental effects, particularly with 3 T MRI, and the implications of gadolinium exposure. While some animal studies have raised concerns about the retention of gadolinium in fetal tissues, clinical studies have not consistently demonstrated adverse effects, with one large cohort study showing no increased risk of neonatal death or significant long-term complications.

The transition to higher field strengths offers enhanced diagnostic capabilities but also raises concerns about potential safety implications, such as increased tissue heating and the effect of higher acoustic noise levels. While preliminary studies on acoustic damage and fetal brain temperature suggest that 3 T MRI is within safety limits, further large-scale investigations are needed to definitively establish its safety, particularly in early pregnancy.

Future research should prioritize comprehensive, large-scale prospective studies with long-term follow-ups and more detailed data collection to better understand the full range of risks, particularly regarding the use of GBCAs and the effects of higher field MRI systems. Additionally, the investigation of fetal MRI in early pregnancy, especially with emerging contrast agents, is crucial for refining safety protocols and ensuring the optimal use of MRI in pregnant patients.
